# Association between the dispositional optimism and depression in young people: a systematic review and meta-analysis

**DOI:** 10.1186/s41155-021-00202-y

**Published:** 2021-11-29

**Authors:** Fabio Alexis Rincón Uribe, Silvia Botelho de Oliveira, Amauri Gouveia Junior, Janari da Silva Pedroso

**Affiliations:** 1 Programa de Pós-Graduação Psicologia, Universidade Federal do Pará, Rua Augusto Corrêa, 01-Guamá, Belém, Pará 66075-110 Brazil; 2 Programa de Psicología, Universidad Pontificia Bolivariana, Bucaramanga, Colombia; 3 Programa de Pós-Graduação em Teoria e Pesquisa do Comportamento, Universidade Federal do Pará, Belém, Brazil; 4 Programa de Pós-Graduação em Teoria e Pesquisa do Comportamento, Universidade Federal do Pará, Belém, Brazil

**Keywords:** Dispositional optimism, Depression, Depressive symptoms, Youth

## Abstract

The objective of this study was to perform a meta-analysis of publications that reported the association between dispositional optimism and depression during youth, analyzing if the strength of this relationship varied according to potential factors. Systematic searches were carried out in APA PsycNet, Virtual Health Library, Embase, Web of Science, PubMed Central, and Scopus to collect English, Portuguese, or Spanish studies from 2009 onwards. Two reviewers selected the eligible articles, assessed the quality of each study, and extracted the data. For the synthesis of the results, a meta-analytic approach was used. We identified 4077 publications in the initial searches and 22 in the supplementary searches, from which 31 studies remained for analysis once the eligibility criteria were applied. The results showed a statistically significant negative association between dispositional optimism and depression in the young population, age being a factor that modifies the effect measure between these variables. This meta-analysis provides a consistent and robust synthesis on the interaction effect between dispositional optimism and depression in the young population. Based on these findings, early clinical admissions may effectively improve optimistic tendencies in young people, which could help them prevent depressive symptoms or episodes.

**Supplementary Information:**

The online version contains supplementary material available at 10.1186/s41155-021-00202-y.

## Introduction

Depression is one of the most common psychopathological conditions in the general population (Global Burden of Disease, 2020), and its prevalence has been increasing in recent decades (Lim et al., 2018). It characterizes by episodes of sadness, emotional emptiness, low self-esteem, feeling tired, insomnia, lack of appetite, anhedonia, and feelings of guilt (Tolentino & Schmidt, 2018), and its most serious state, self-harm (Murphy et al., 2010) or suicide (Orsolini et al., 2020) can occur. It shows a tendency for a chronic recurrent course of the disease with high levels in the clinical population and lower levels in the general population (Hardeveld et al., 2010), having its onset and development during adolescence or early adulthood (Wilson et al., 2015).

Due to its impact on society, understanding depression has been a demanding task in psychological research, which has led researchers to formulate explanatory frameworks for depression. One of the predominant theories is the cognitive model, which indicates that the acquisition and processing of a series of negative, biased, and maladaptive cognitions influence the development and maintenance of depression (Beck & Haigh, 2014). Although scientists added in subsequent expansions of this model, relevant behavioral, genetic, evolutionary, psychosocial, and neurobiological variables (Beck, 2008; Beck & Bredemeier, 2016), recently, the study of expectations has also played a crucial role in understanding depressive experiences in the general population (Horwitz et al., 2017; Kube et al., 2017; Zetsche et al., 2019). Within this group, positive expectations, operationalized under the construct of dispositional optimism, represent a particular type of expectations negatively associated with depression (Chung et al., 2016; Wrosch et al., 2017).

Within the construct of dispositional optimism, positive expectations represent the most relevant cognitive factor in assessing the extent to which future outcomes are perceived as good (Carver & Scheier, 2001). From this perspective, dispositional optimism is a facet of personality that reflects the extent to which people have generalized positive expectations for their future (Carver & Scheier, 2014). This perspective is related to the expectancy-value theory of achievement motivation, which holds that behavior is an expression of the search for goals, states, or desired actions (Wigfield, 1994). According to this approach, more optimistic and motivated individuals exert more effort, whereas pessimists refrain from such efforts (Carver et al., 2010). In the clinical context, dispositional optimism has become relevant due to its high association with psychopathological risk or the adoption of healthy behaviors (Kleiman et al., 2017; Sardella, Lenzo, Bonanno, Basile, & Quattropani, 2021). For example, greater optimism is inversely related to risk factors for depression such as hopelessness or rumination (Fischer et al., 2018; Tucker et al., 2013). On the other hand, the most optimistic individuals appear to improve their resilience, which may give them a better ability to adapt to stressful or traumatic events and a lower risk of relapse (Sardella, Lenzo, Bonanno, Martino, et al., 2021).

In recent decades, empirical advances focused on exploring the effect of dispositional optimism on various mental health outcomes in people have provided substantial evidence for understanding depression (Carver et al., 2010). Such contributions have transcended into the clinical practice and treatment of subjects with different levels of depression (Karlsson et al., 2011). Being positive expectations the central measure of dispositional optimism (Carver & Scheier, 2014), previous evidence has reported that people who are more optimistic about their future have lower levels of depression (Ironson et al., 2005; Tindle et al., 2012) prospectively reducing the risk of experiencing the severity of depressive symptoms, such as anhedonia, dysphoria, or sadness (Boelen, 2015; Kleiman et al., 2017).

From an epidemiological perspective, adolescence and early adulthood are considered critical periods for the development of depressive episodes and symptoms (Kessler & Bromet, 2013); however, the literature suggests that the tendency toward optimism may also originate in this phase. Accordingly, dispositional optimism might originate in adolescence, where it shows a linear pattern that is positively associated with age and tends to increase until emerging adulthood (Renaud et al., 2019; Zou et al., 2016). In the literature, some studies have shown that more optimistic adolescents have fewer symptoms or depressive episodes (Dooley et al., 2015; Sing & Wong, 2011) and that this dispositional optimism could serve as an indicator for depression prevention (Piko et al., 2013; Wang et al., 2012). Although these findings suggest that dispositional optimism has an important effect in reducing and preventing depression at these stages of human development, these results do not analyze whether this effect might differ as a function of age.

The study of the cognitive factors associated with depression has focused mainly on the understanding of negative cognitions. There is a significant scientific interest in exploring how positive variables such as dispositional optimism could explain depressive symptoms or episodes, especially in critical stages such as youth. Therefore, synthesizing the evidence on the relationship between dispositional optimism and depression is an essential step in understanding the effects or impact of positive future-focused expectations on depressive experiences in young people. The objective of this systematic review and meta-analysis was to examine the evidence between January 2009 and August 2020 to have a better understanding of the relationship between these variables. According to previous evidence, this systematic review and meta-analysis questions were: What is the association between dispositional optimism and depression in young people? Is there a potential factor that modifies the association between dispositional optimism and depression?

## Method

### Study design

We performed this systematic review and meta-analysis according to the parameters The PRISMA 2020 Statement: an updated guideline for reporting systematic review (PRISMA) (Page et al., 2021). The review protocol was registered in the International Prospective Registry of Systematic Reviews–PROSPERO (Registry number: CRD42020200455).

### Eligibility criteria

This review included empirical quantitative studies based on cross-sectional or longitudinal designs, available in full text and published in English, Portuguese, or Spanish. Due to the growing research on dispositional optimism in the clinical context, we limited the range of publication years from January 2009 to August 2020. Participants were young people between 10 and 25 years old (adolescents were between 10 and 19 years old, and young adults were between 20 and 25 years old) (World Health Organization, 2011). In this way, we included studies with samples exposed to validated evaluation measures for dispositional optimism and depression, and their main results presented a relevant effect measure for the analysis. Studies that did not provide sufficient original data and gray literature manuscripts (Dissertations, books, chapters, commentary, reports, conference material, opinion piece) were excluded.

### Search strategy

The searches were carried out from August 2020 to December 2020 in the following electronic databases: APA PsycNet (American Psychological Association), BVS (Virtual Health Library), Embase, Web of Science (Science and Social Science Citation Index), PubMed Central, and Scopus. For the search strategy, controlled MeSH (Medical Subject Headings) vocabulary was included, such as “optimism,” “depression,” “depressive symptoms,” “youth,” “adolescent,” and “young adult.” The search terms were tested and adjusted for all databases, using the Boolean operators and other descriptors associated with the MeSH terms. Details of the search strategy are available in supplementary material. After preliminary searches of the databases, the authors manually searched on Academic Google and checked the reference lists of studies relevant to the review.

### Study selection

All bibliographic citations were imported to Mendeley software for the organization of the articles and the elimination of duplicates. Independently, the reviewers performed an initial selection of the literature, extracting potentially relevant studies by titles and abstracts. Subsequently, the authors applied the inclusion and exclusion criteria independently for the full-text evaluation and the final inclusion of the relevant articles in the study. The reviewers discussed any discrepancies generated during the process to reach a consensus.

### Data extraction

One reviewer extracted data with complete verification of the second reviewer, using a standardized extraction form previously designed in Excel. The following data were extracted: country, type of study, general objective, characteristics of the participants, methods of each study (design and analysis methods), outcome measures (measuring instruments), primary outcomes (measures of effects–*r* weighted). After data extraction, reviewers made consensus through debates.

### Methodological quality assessment

The methodological evaluation of the included studies was carried out using the version adapted for cross-sectional and longitudinal designs of the Newcastle-Ottawa Scale (NOS) (Wells et al., 2011; Herzog et al., 2013). The NOS has an additive rating system based on three evaluation criteria: (1) Selection (representativeness of study participants); (2) Comparability (determination of confounding factors); and (3) Result (evaluation and analysis of results). The grade is assigned as absent (0) or present (1 or 2), and its sum gives an evaluation score between 0 to 10 points. Since the NOS does not provide clearly defined and standardized cut-off points, the scores were established considering previously published studies (Modesti et al., 2016; Tomczyk et al., 2016). Thus, we classified the methodological quality as high (10 and 9 points), medium (7 and 8 points), or low (< 7 points). In general, the NOS has adequate reliability in observational studies (Margulis et al., 2014).

### Data analysis and synthesis

In this review, we used a meta-analytical approach to analyze and synthesize data. All statistical analyses and graphics were performed using the R software (Version 4.0.5) and the meta and metacor R packages to calculate the values (Harrer et al., 2019). The meta-analysis was carried out by calculating Fisher’s *Z* values to obtain the weights of each study and combining the reported weighted *r* in a pooled correlation estimate through the generic inverse variance combination method (Lipsey & Wilson, 2001). The heterogeneity of the studies was evaluated using the Cochrane *Q* statistic with a level of statistical significance set at *p* < 0.01, and the magnitude using the *I*^2^ statistic, the result of which was interpreted using the following classification: low (25% - 49%,), moderate (50–74%), and high (≥ 75%) heterogeneity, with a significance level of *p* < 0.01 (Higgins et al., 2003).

Considering that significant heterogeneity was expected in the studies, we used the random-effects model to derive effect sizes and confidence intervals. The random-effects models are based on the assumption of variation in the effect size between the studies due to sampling errors and the actual random variance based on the differences in methods and application contexts. These models, unlike fixed models, are considered preferable for generalization and unconditioned inference beyond the current group of studies to other similar ones that could be carried out (Schmid et al., 2009). In addition, because the use of the random effects model was expected, the variance of the heterogeneity of the actual effects of the studies was estimated using the tau-squared (*T*^2^) (Borenstein et al., 2009). The Sidik-Jonkman estimator was used calculate *T*^2^ (Sidik & Jonkman, 2005).

Additionally, exploratory subgroup analyses were performed based on the following characteristics: age (adolescents and young adults), ethnic origin (continent), evaluation measures (types of measures for depression), and measurement context (academic and general population). We used the random-effects model to combine the studies within the established subgroups and calculated the *Q* statistic to compare the effect sizes detected in each subgroup, with a significance level for this test of *p* < 0.05 (Richardson et al., 2019). We perform a sensitivity analysis of leaving one out by skipping one study at a time to determine whether or not the results are dependent on one study. The publication bias of the studies was examined using funnel plots and Egger’s intersection test with a significance level of *p* < 0.05 (Egger et al., 1997).

## Results

**Fig. 1:**
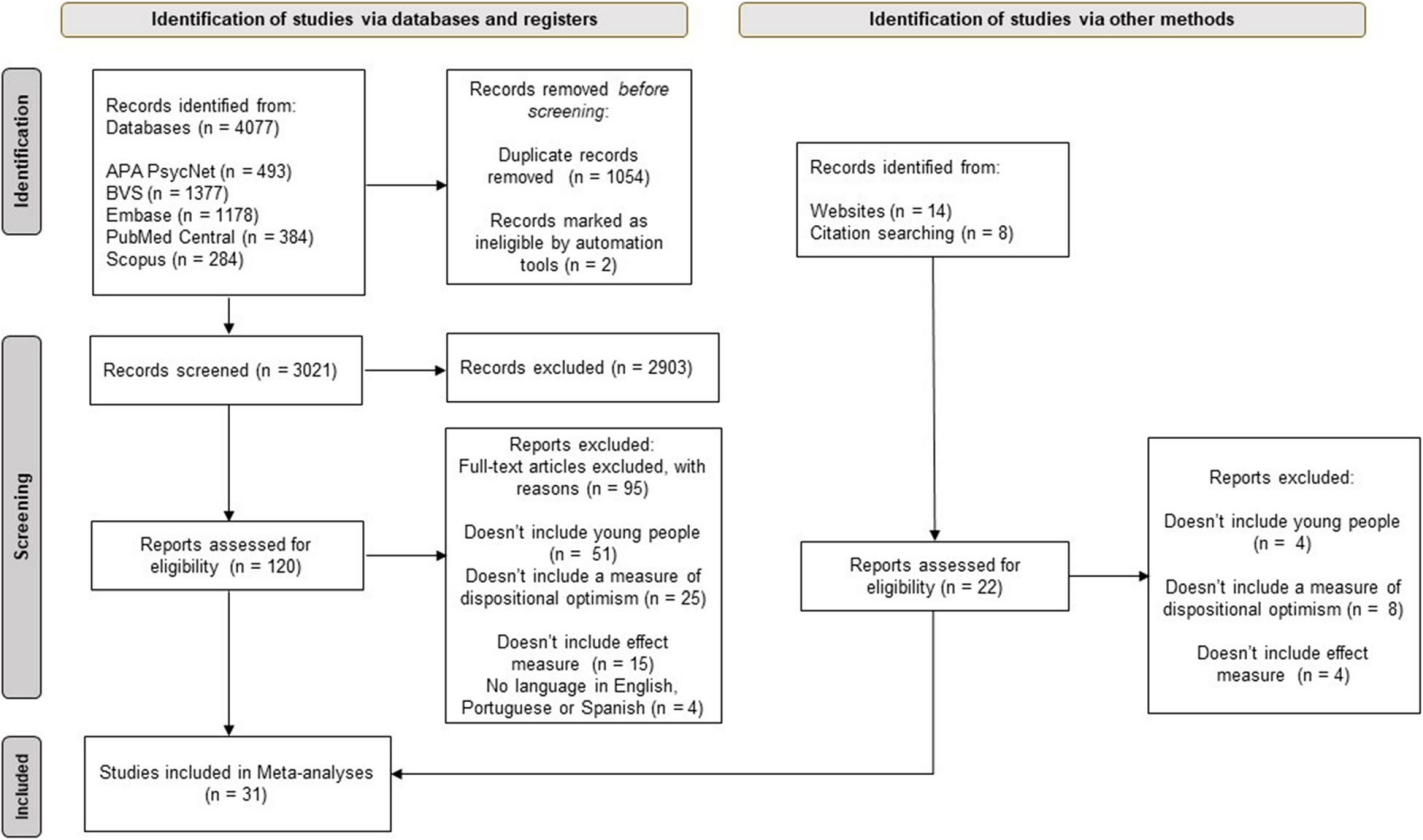
PRISMA flow chart. Detailed description of the study selection process through the different phases of a systematic review. The number of records identified, included and excluded, as well as the reasons for exclusions are indicated

We identified a total of 4077 publications in the total searches of the online databases. In addition, we found 22 relevant studies using hand searches by checking reference lists and complementary searches on websites. After excluding duplicates and refining the search results by selecting titles and abstracts, 120 articles remained for the full-text selection. Finally, when applying the eligibility criteria, 49 were excluded because they did not include representative samples of young people, 32 because they did not include measures of dispositional optimism, 19 because they did not analyze relevant effect measures for the analysis, and 4 because the language was different from English, Portuguese, or Spanish, leaving 31 articles eligible for the final synthesis. Figure 1 presents the detailed study selection process using the PRISMA flow chart.

### Characteristics of the studies

In total, 31 relevant studies that evaluated the correlation between dispositional optimism and depression in young people, with a publication date ranging between 2009 and 2020, remained for analysis. These studies evaluated population cohorts from Australia, Canada, China, Germany, Hong Kong, Hungary, Ireland, Pakistan, Romania, Singapore, Turkey, and USA. The total number of participants was 26,325, of which 6656 were adolescents aged between 10 and 18 years and 19,659 were young adults aged between 19 and 25 years. Of the total of studies, 29 reported cross-sectional data analyses, and 2 analyzed longitudinal data.

For the measure of dispositional optimism, 93.3% of the studies used the revised, short, or extended version of the Life Orientation Test; 3.3% used the Self-Efficacy, Optimism, and Pessimism Instrument (SWOP); and 3.3% used Adolescents’ Optimism Scale. Regarding the measure of depression, 29% (*n* = 9) of the studies used the Center for Epidemiologic Studies Depression Scale (CES-D), 12.9% (*n* = 4) used the Beck Depression Inventory (BDI), 12.9% (*n* = 4) applied the Beck Depression Inventory-II (BDI-II), 6.5% (*n* = 2) used the Depression, Anxiety, and Stress Scale (DASS-21), 6.5% (*n* = 2) used the Self-Rating Depression Scale (SDS), 6.5% (*n* = 2) used the Childhood Depression Inventory (CDI), 6.5% (*n* = 2) used the Brief Symptoms Inventory (BSI), 6.5% (*n* = 2) used the Hospital Anxiety and Depression Scale (HADS), 3.2% (*n* = 1) applied the Reynolds Adolescent Depression Scale (RADS-2), 3.2% (*n* = 1) used the Southern Child and Adolescent Mental Health Service Depression and Anxiety Scale (SCAMDA), 3.2% (*n* = 1) used the Revised Children's Anxiety and Depression Scale–Short Version ( RCADS-SV), and 3.2% (*n* = 1) used the Patient Health Questionnaire for Depression and Anxiety (PHQ-4). The details of the studies are in Table 1.

**Table 1:** Characteristics of the included studies

Author, year	Country, continent	Design	Context	Sample size	Mean age ± SD, (range)	Outcome measures	
						Dispositional optimism	Depression
Chang et al., 2018	Hungary, Europe	Cross-sectional	Academic	457	21.52 ± 2.16, (18–32)	LOT-R	BDI
Chin & Holden, 2013	Canada, America	Cross-sectional	Academic	87	18.15 ± 0.84, (18–23)	LOR-R	BDI-II
Cobb et al., 2020	United State, America	Cross-sectional	General population	200	21.30 ± 2.09	LOT-R	CES-D
Finch et al., 2020	Australia, Oceania	Cross-sectional	Academic	486	11.54 ± 1.20.	YLOT	RCADS-SV
Guassi & Telzer, 2015	United State, America	Longitudinal	Academic	338	18.40 ± 0.36	LOT-R (T1)	CES-D (T1)
						LOT-R (T1)	CESD (T2)
Heinen et al., 2017	Germany, Europe	Cross-sectional	Academic	321	21.80 ± 3.93	SWOP	PHQ-4
Hinkle & Quiton, 2019	United State, America	Cross-sectional	Academic	77	22.99 ± 6.81	LOT-R	BDI
Iovu et al., 2018	Romania, Europe	Cross-sectional	General population	1509	(18–25)	LOT-R	BDI
Jiang et al., 2015	China, Asia	Cross-sectional	Academic	1200	20.73 ± 1.37	LOT-R	SDS
Kaiser & Malik, 2015	Pakistan, Asia	Cross-sectional	Academic	400	16.0 ± 1.6, (14–18)	LOT-R	DASS
Kapıkıran & Acun-Kapıkıran, 2016	Turkey, Asia	Cross-sectional	Academic	494	20.85 ± 1.57, (18–30)	LOT-R	BSI
Kenny et al., 2016	Ireland, Europe	Cross-sectional	Academic	8121	20.42 ± 1.90, (17–25)	LOT-R	DASS
Kube, Glombiewski, & Rief, 2018	Germany, Europe	Longitudinal	Academic Academic	125	22.05 ± 4.00	LOT-R (T1)	BDI-II (T1)
						LOT-R (T2)	BDI-II (T2)
Kwok & Gu, 2017	Hong Kong, Asia	Cross-sectional	Academic	525	13.80, (12–15)	LOT-R	HADS
Kwok & Gu, 2019	Hong Kong, Asia	Cross-sectional	Academic	302	10.56, (9–12)	LOT-R	BSI
Liu & Lau, 2013	United State, America	Cross-sectional	Academic	670	18.92 ± 1.08, (17–22)	LOT-R	CES-D
Liu et al., 2017	China, Asia	Cross-sectional	Academic	1205	19.86 ± 1.26, (17–24)	LOT-R	CES-D
Morris et al., 2010	United State, America	Cross-sectional	Academic	235	18,00 ± 2.00	LOT-R	CES-D
Morton et al., 2014	Australia, Oceania	Cross-sectional	Academic	84	(17–18)	LOT-R	SCAMDA
Niu et al., 2018	China, Asia	Cross-sectional	Academic	1626	14.31 ± 1.52, (12–18)	LOT-R	CES-D
O'Sullivan et al., 2019	Canada, America	Cross-sectional	General population	886	20.7, (18–22)	LOT-R	BDI-II
Pinquart & Pfeiffer, 2014	Germany, Europe	Cross-sectional	Academic	162	16.9 ± 2.6	LOT-R	CDI
Pu et al., 2017	China, Asia	Cross-sectional	Academic	535	20.67 ± 1.43, (18–23)	LOT-R	SDS
Shi et al., 2016	China, Asia	Cross-sectional	Academic	2925	21.65 ± 1.95, (15–28)	LOT-R	CES-D
Tyser et al., 2014	United State, America	Cross-sectional	Academic	164	Age not informed	LOT-R	CDI
Wang et al., 2018	China, Asia	Cross-sectional	Academic	234	18.60 ± 0.78	LOT-R	BDI
Weber et al., 2010	United State, America	Cross-sectional	Academic	179	15.6, (14-18)	LOT-R	RADS-2
Wong & Lim, 2009	Singapore, Asia	Cross-sectional	Academic	334	15.6 ± 0.59, (14–18)	LOT-R	CES-D
Wu et al., 2015	China, Asia	Cross-sectional	General population	50	22.2 ± 1.7, (19–25)	LOT-R	BDI-II
Xie et al., 2018	China, Asia	Cross-sectional	Academic	1742	14.35 ± 1.52)	AOS	CES-D
Zou et al., 2020	China, Asia	Cross-sectional	Academic	652	14.55 ± 1.82, (1120)	LOT-R	CES-D

*AOS* Adolescents Optimism Scale, *BDI* Beck Depression Inventory, *BDI*-II Beck Depression Inventory–II, *BSI* Brief Symptom Inventory, *CDI* Children’s Depression Inventory, *CES*-*D* Center for Epidemiologic Studies Depression Scale, *DASS* Depression Anxiety and Stress Scale, *HADS* Hospital Anxiety and Depression Scale, *LOT*-*R* Life Orientation Test-Revised, *PHQ*-*4* Patient Health Questionnaire for Depression and Anxiety, *RADS*-*2* The Reynolds Adolescent Depression Scale, *RCADS*-*SV* Revised Children’s Anxiety and Depression Scale–Short Version, *SCAMDA* Southern Child and Adolescent Mental Health Service Depression and Anxiety Scale, *SD* Standard Deviation, *SDS* Self-Rating Depression Scale, *SWOP* Self-Efficacy, Optimism and Pessimism Instrument, *YLOT*-*R* Young Life Orientation Test-Revised

### Assessment of the methodological quality of the studies

Overall, 18 (58%) of the included studies presented moderate methodological quality, while 8 (26%) and 5 (16%) showed low and high qualities, respectively. All the studies with moderate methodological quality included the validation instruments and analyzed two or more confounding factors relevant to the analysis of the results (Chang et al., 2018; Cobb et al., 2020; Finch et al., 2020; Guassi & Telzer, 2015; Heinen et al., 2017; Kapıkıran & Acun-Kapıkıran, 2016; Kwok & Gu, 2017; Kwok & Gu, 2019; Liu & Lau, 2013; Liu et al., 2017; Niu et al., 2018; O'Sullivan et al., 2019; Pinquart & Pfeiffer, 2014; Pu et al., 2017; Shi et al., 2016; Tyser et al., 2014; Wang et al., 2018; Wu et al., 2015). However, 10 studies did not include representative samples in relation to the target population (Chang et al., 2018; Cobb et al., 2020; Finch et al., 2020; Heinen et al., 2017; Kapıkıran & Acun-Kapıkıran, 2016; Kwok & Gu, 2017; Kwok & Gu, 2019; Tyser et al., 2014; Wang et al., 2018; Wu et al., 2015).

**Fig. 2:**
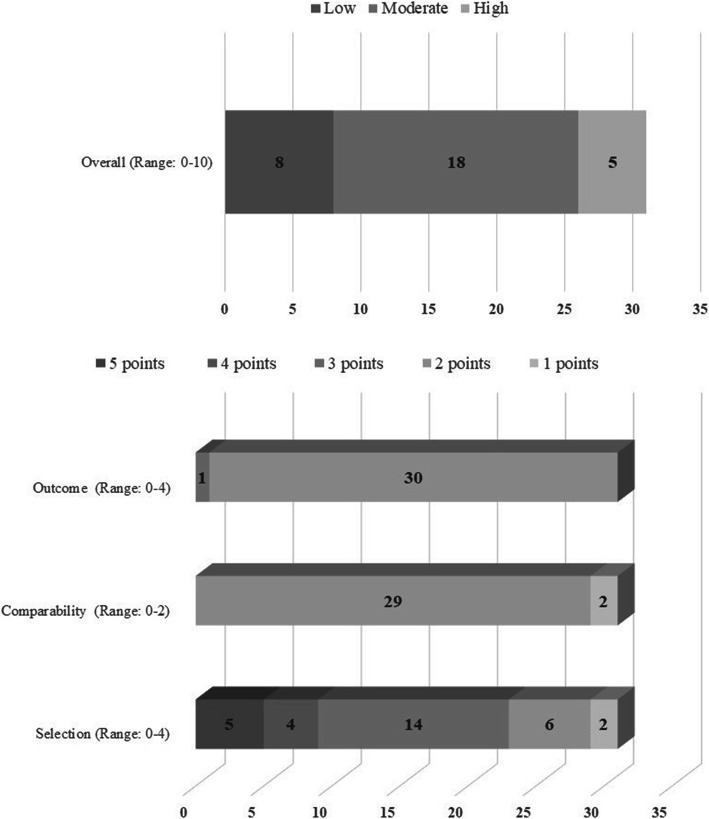
Results of the assessment of the methodological quality of studies using NOS. The graph shows the general ang criteria scores of the studies. The interpretation of these results is made according to the following range: high (10 and 9 points), moderate (7 and 8 points), or low (< 7 points) methodological quality

On the other hand, all the studies with low methodological quality were characterized by having non-representative samples in relation to the target population and using non-probabilistic sampling to select participants (Chin & Holden, 2013; Hinkle & Quiton, 2019; Kaiser & Malik, 2015; Kube, Anna, et al., 2018; Morris et al., 2010; Morton et al., 2014; Weber et al., 2010; Wong & Lim, 2009). Likewise, 2 studies did not report the validation of the measurement instruments (Hinkle & Quiton, 2019; Kaiser & Malik, 2015) and 2 analyzed only one confounding factor relevant to the analysis of the data (Morris et al., 2010; Weber et al., 2010). The total scores and those of each criterion proposed by the NOS appear in Fig. 2.

### General meta-analysis: association between dispositional optimism and depression

**Fig. 3:**
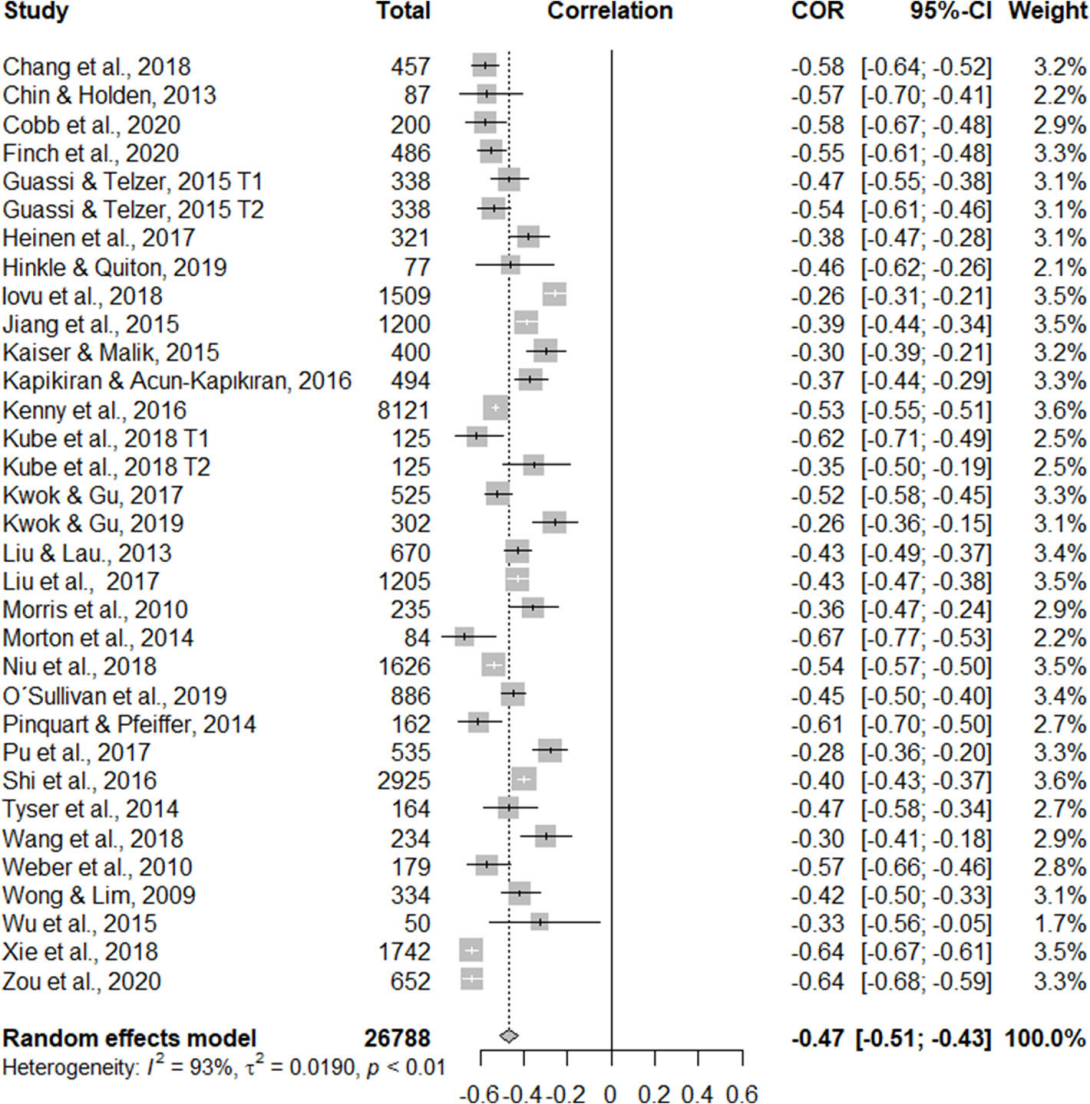
Forest plot of the overall meta-analysis. The weighted effect size corresponds to the overall association between dispositional optimism and depression in young people

The general meta-analysis of the studies presented a significant heterogeneity of *I*^2^ = 93%, *T*^2^ = 0.0109, *p* < 0.01, which indicates that the use of the random effects model is adequate. The results presented a significant negative effect between dispositional optimism and depression (*r* = − 0.47, 95% CI = − 0.51, − 0.43) in the general population of young people. The effect sizes, confidence intervals (95%), and each study’s weight are in the forest plot (see Fig. 3).

### Subgroup analysis

Table 2 shows the results of the subgroup analysis of the studies that reported effect sizes on the association between dispositional optimism and depression in the young population.

**Table 2:** Results of subgroup analysis

Category	Subgroup	Studies (*f*)	*N*	Combined correlation (95% IC)	*p* value
Age	Adolescent	12	6656	− 0.52 [− 0.59; − 0.45]	
	Young adults	19	19669	− 0.44 [− 0.48; − 0.39]	
	Total	31			*0.046*
Continent	America	9	2836	− 0.49 [− 0.53; − 0.44]	
	Asia	13	11730	− 0.43 [− 0.51; − 0.36]	
	Europa	7	11189	− 0.47 [− 0.56; − 0.37]	
	Oceania	2	570	− 0.60 [− 0.70; − 0.47]	
	Total	31			*0.178*
Context	Academic	27	23680	− 0.48 [− 0.52; − 0.43]	
	General Population	4	2645	− 0.41 [− 0.55; − 0.26]	
	Total	30			*0.407*
Measure of depression	BDI	4	2277	− 0.40 [− 0.55; − 0.24]	
	BD− II	3	1148	− 0.48 [− 0.58; − 0.36]	
	BSI	2	1164	− 0.40 [− 0.46; − 0.34]	
	CDI	2	326	− 0.54 [− 0.66; − 0.40]	
	CES− D	9	9257	− 0.51 [− 0.57; − 0.44]	
	DASS	2	8521	− 0.43 [− 0.62; − 0.18]	
	HADS	2	827	− 0.40 [− 0.62; − 0.13]	
	SDS	2	1735	− 0.34 [− 0.44; − 0.23]	
	Total	25			*0.088*

*BDI* Beck Depression Inventory, *BDI*-II Beck Depression Inventory–II, *BSI* Brief Symptom Inventory, *CDI* Children’s Depression Inventory, *CES*-*D* Center for Epidemiologic Studies Depression Scale, *CI* confidence interval, *DASS* Depression Anxiety and Stress Scale, *HADS* Hospital Anxiety and Depression Scale, *SDS* Self-Rating Depression Scale

#### Age

The test for subgroup differences by age indicates that there is a statistically significant effect (*Q* = 3.96; d.f = 1 *p* = 0.046); this suggests that age significantly modifies the effect size between dispositional optimism and depression, being higher in adolescents with *r* = − 0.52 [95% CI: − 0.59, − 0.44] than in young adults with *r* = − 0.43 [95% CI: − 0.48, − 0.38].

#### Continent

Across continents, the pooled correlation between dispositional optimism and depression for the total population was highest in Oceania (*n* = 570) with *r* = − 0.60 [95% CI: − 0.70; − 0.47], followed by America (*n* = 2836) with *r* = − 0.49 [95% CI: − 0.53; − 0.44], Europe (*n* = 11,189) with *r* = − 0.47 [95% CI: − 0.56; − 0.37], and Asia (*n* = 11730) with *r* = − 0.43 [95% CI: − 0.51; − 0.36]. However, these differences were not statistically significant (*Q* = 4.91; d.f = 3; *p* = 0.178).

#### Context

Studies conducted in academic settings (*n* = 23,680) had a higher combined correlation between dispositional optimism and depression (*r* = − 0.48 [95% CI: − 0.52, − 0.43]) than those performed in the general population (*n* = 2645; *r* = − 0.41 [95% CI: − 0.55, − 0.26]). This difference was not statistically significant (*Q* = 0.69; d.f = 1; *p* = 0.413).

#### Depression measure

Regarding the measure of depression, the studies that used the Children’s Depression Inventory (CDI) and the Center for Epidemiologic Studies Depression Scales (CES-D) obtained the highest effect sizes (*r* = − 0.54 [CI of 95%: − 0.66; − 0.40] and *r* = − 0.51 [CI of 95%: − 0.57; − 0.44], respectively). Then we find the Beck Depression Inventory-II (BD-II) with (*r* = − 0.48 [CI of 95%: − 0.58; − 0.36]), the Depression Anxiety and Stress Scale (DASS) with (*r* = − 0.43 [ 95% CI: − 0.62, − 0.18]), the Brief Symptom Inventory (BSI) (*r* = − 0.40 [95% CI: − 0.46, − 0.34]), the Hospital Anxiety and Depression Scale (HADS) (*r* = − 0.40 [95% CI: − 0.62; − 0.13]), the Beck Depression Inventory (BDI) (*r* = − 0.40 [95% CI: − 0.55; − 0.24]), and finally the Self-Rating Depression Scale (SDS) (*r* = − 0.34 [95% CI: − 0.44, − 0.23]). The difference was not statistically significant (*Q* = 12.41; d.f = 7; *p* = 0.088).

### Publication bias

**Fig. 4:**
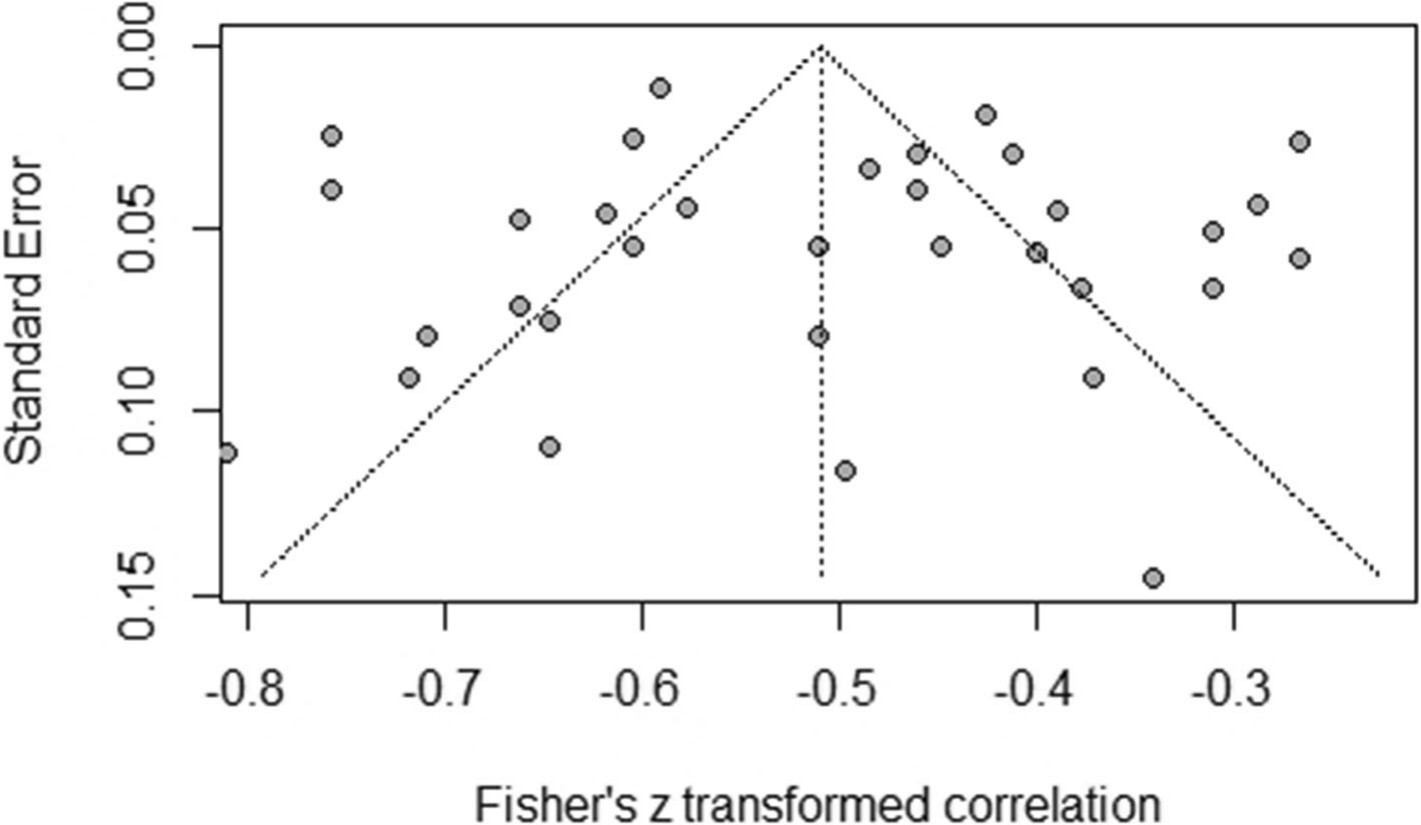
Begg’s funnel plot for publication bias of general meta-analysis. Begg’s funnel plot for publication bias on the relationship between dispositional optimism and depression in young people. The standard error of the transformed effect size is shown on the y-axis, and the pooled Fischer *Z*-values are presented on the *x*-axis

In the evaluation of publication bias of the studies, the funnel plot shows a degree of asymmetry (see Fig. 4). In Egger’s test, an intercept value of 0.986 [95% CI: − 1.366; − 3,338; *t* = 0.825] with a *p* value of 0.415, indicating that there is no substantial asymmetry in the funnel plot and therefore there is no evidence of publication bias.

### Sensitivity analysis

**Fig. 5:**
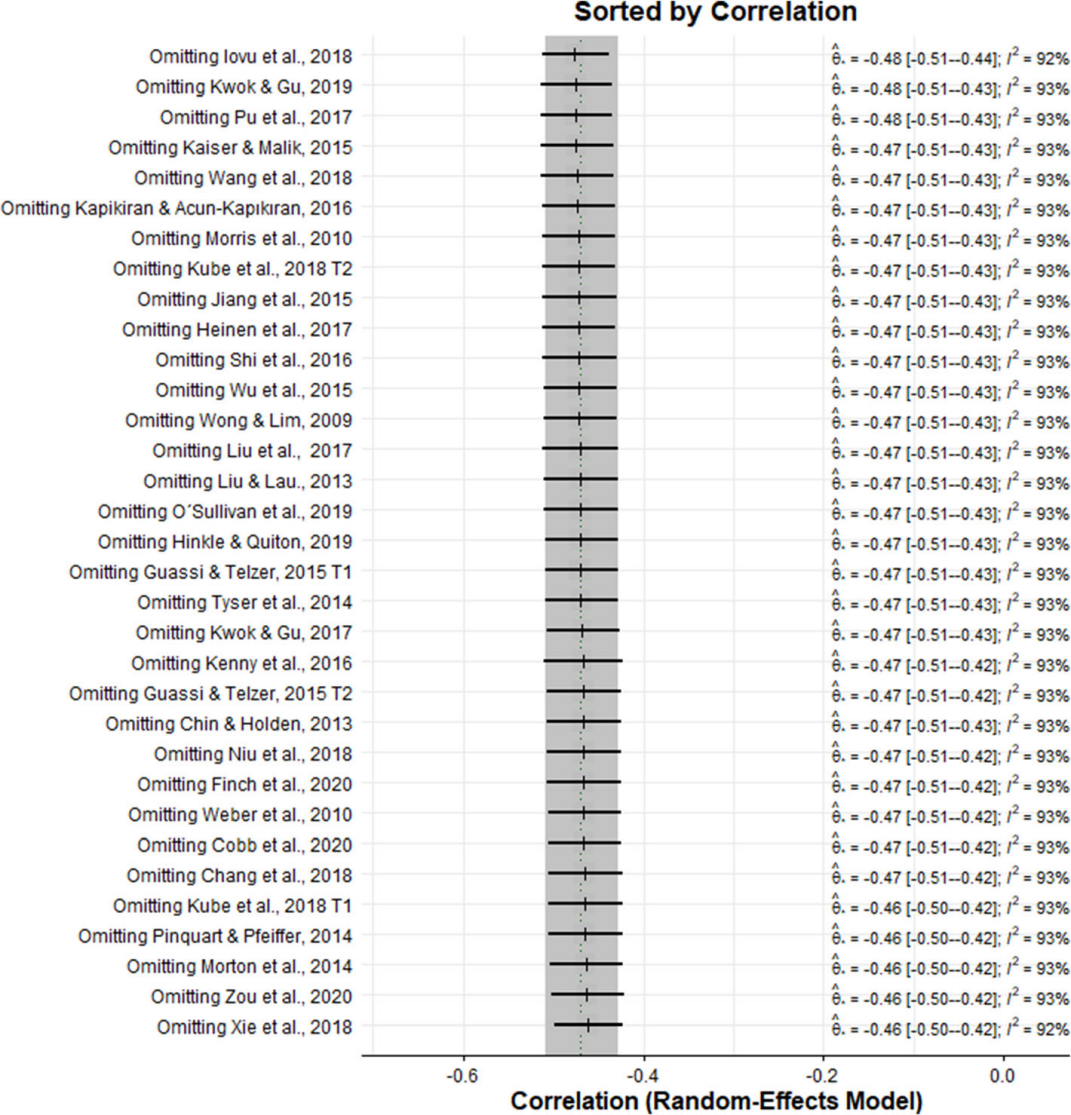
Results of the sensitivity analysis of leaving one out. Sensitivity analysis on the correlation between dispositional optimism and depression. The result that leaving out a study is represented by a combined correlation estimate with 95% CIs

In this meta-analysis, a sensitivity analysis of leaving one out was performed by removing one study at a time and recalculating the combined correlation. Between the studies, the combined effect measure did not vary substantially with a range between − 0.48 [CI − 0.51; − 0.44] and − 0.46 [− 0.50; − 0.42], which indicates that the results were stable and do not depend on a single study (see Fig. 5).

## Discussion

This review identified 31 eligible studies that examined the association between dispositional optimism and depression in young people. In general, the results of this meta-analysis are consistent with the literature, indicating a statistically significant negative cross-sectional relationship between dispositional optimism and depression in the young population (Ling et al., 2016; Wang et al., 2012). Consistent with current cognitive models of depression, people with low levels of dispositional optimism have more dysfunctional or harmful expectations about their future, leading to more severe depressive symptoms (Kube et al., 2018). These results support the idea that positive expectations could explain the occurrence or prognosis of depressive symptoms in young people samples (Vickers & Vogeltanz, 2000). However, according to the overall result of the meta-analysis (*r* = − 0.47, *r*^2^ = 0.22), there is 78% of variance that is not explained by optimism. This finding indicates that other variables such as education, social relationships, parents, or family background could explain significant variance in depression.

In this meta-analysis, the relationships found between optimism and depression were independent of ethnic origin, context, and measures used to evaluate depression, but not for age; this would indicate an association that could not be constant throughout human development. Although there are no differences by age in the clinical or diagnostic pictures of depression (Lewinsohn et al., 2003; Thapar et al., 2012), the prevalence of depressive episodes is higher in adolescents than in young adults, maintaining significant growth from 12 to 20 years (Mojtabai et al., 2016). The relationships between dispositional optimism and depression obtained a more powerful correlation in adolescents than in young adults, suggesting that positive expectations could play a more critical role in depressive episodes during adolescence. Therefore, it would be necessary to consider sociodemographic factors, such as age and its relationship with dispositional optimism in their effect on depression during the stages of human development.

Why is dispositional optimism important in research or clinical treatment of depression in youth? Previous studies have indicated that young people with a more optimistic tendency about their future present more behaviors related to good health (Klimusová et al., 2016) and less risk of experiencing psychopathological symptoms (Xiong et al., 2020). Being dispositional optimism a cognitive resource whose origin could occur during adolescence (Zou et al., 2016), some evidence has suggested that a more optimistic orientation in young people could be an essential health asset (Häggström et al., 2017), which could function as a protective factor against psychopathological conditions such as depression (Ames et al., 2015). Empirically, it has been shown that dispositional optimism during adolescence is associated with a better perception of internal locus of control and this, in turn, is related to more adaptive self-regulation (Renaud et al., 2019). This adaptive self-regulation implies that optimistic people continually focus on fulfilling their objectives, thus reducing their participation in risky situations and giving more meaning to life (Carver & Scheier, 2001).

Although this meta-analysis presented a significant heterogeneity index in the pooled data, we found a methodological homogeneity in using instruments to evaluate dispositional optimism, being the Life Orientation Test-Revised (LOT-R), the outcome measure most used by the authors. According to the literature, the LOT-R is a frequently used measure due to its stability and reliability in measuring dispositional optimism (Hinz et al., 2017; Scheier & Carver, 2018), both in physical and mental health contexts (Schou et al., 2017). Thus, in this meta-analysis, we found a statistically significant cross-sectional relationship between dispositional optimism, as measured by the LOT-R, and depression in the young population. Due to the increasing adaptability of the LOT-R to different dimensions of health, its application could provide efficient and simplified indicators that would help therapists or researchers to predict the occurrence of symptoms associated with depression in people (Karlsson et al., 2011). In light of this evidence, the application of the LOT-R is recommended in both community and clinical samples, being able to function as a reliable psychometric indicator in the early detection of depressive symptoms or episodes.

### Clinical implications

Dispositional optimism has a negative association with depressive symptoms and episodes in the young population. Although these results do not offer longitudinal evidence on the impact of dispositional optimism on developing depressive disorders in youth, carrying out early interventions based on optimism could offer young people the opportunity to deal with the symptoms and depressive episodes. It is difficult to recommend a specific intervention. However, it is advisable and pertinent that therapists focus on improving the optimistic tendency of young people since, due to their state characteristics, optimism could change according to circumstances, health, transitions of life, or psychological interventions (Millstein et al., 2019). Some evidence has suggested that the prospective induction of positive thoughts focused on the future can increase the levels of optimism in people, which could significantly reduce the risk of presenting symptoms or depressive episodes (Ji et al., 2017; Miranda et al., 2016).

### Strengths and limitations of the review and meta-analysis

It is necessary to consider the evidence found in this review and meta-analysis based on its strengths and limitations. First, many relevant studies were collected, which allowed the pooling of data drawn from a large sample of the young population. That reflects that the results were based on adequate search strategies and selection criteria for relevant studies. Second, a robust analysis was obtained in the general meta-analysis since the sensitivity analysis showed that the magnitude and direction of the effect remained constant between the studies. Finally, the included studies used validated and reliable measures to obtain their results, finding remarkable homogeneity in the measurement of dispositional optimism. A notable limitation was the high heterogeneity found in the overall meta-analysis, mainly due to variations in effect sizes and the number of participants between the included studies. However, despite this variance, the Eggers test results of the calculation of publication bias support the reliability of the results. Another limitation was the grouping of studies presented under an exclusively cross-sectional design, which made it impossible to examine the cause-and-effect relationship of the variables.

## Conclusions

This review provides an exhaustive synthesis of studies that show a statistically significant negative association between dispositional optimism and depression in the young population, age being a relevant factor that modifies the effect size between these variables. Indeed, positive expectations focused on the future could play an essential role in the development and prevalence of depression in young people, having notable importance during adolescence. Thus, the early improvement of optimistic tendencies toward the future could have relevant effects in preventing symptoms and depressive episodes or promoting well-being in young people.

## Supplementary Information

**Additional file 1.**

## Data Availability

The datasets used and/or analyzed during the current study are available from the corresponding author on reasonable request.
