# Supplementary material for: Association between the dispositional optimism and depression in young people: a systematic review and meta-analysis
Source: Psicol Reflex Crit. 2021 Nov 29;34:37. doi: 10.1186/s41155-021-00202-y (PMC8630239; doi:10.1186/s41155-021-00202-y)
Supplement: Supplementary file 1 — Additional file 1. [file 41155_2021_202_MOESM1_ESM.docx]

| *Keywords* | *Numeration* | *Search criteria* |
| --- | --- | --- |
| Optimism | 1. | Optimism* |
|  | 2. | MeSH descriptor Optimism (this term only) |
|  | 3. | Dispositional optimism |
|  | 4. | #1 OR #2 OR #3 |
| Youth | 5. | Youth* |
|  | 6. | MeSH descriptor Youth (this term only) |
|  | 7. | Adolescent* |
|  | 8. | MeSH descriptor Adolescent (this term only) |
|  | 9. | Young Adult |
|  | 10. | MeSH descriptor Young Adult (this term only) |
|  | 11. | #4 OR #5 OR #6 OR #7 OR #8 OR #9 |
|  | 13. | #4 AND # 10 |
| Depression | 14. | Depression |
|  | 15. | MeSH descriptor depression (this term only) |
|  | 16. | Depressive Symptoms |
|  | 17. | MeSH descriptor Depressive Symptoms (this term only) |
|  | 18. | #14 OR #15 OR #16 OR #17 |
|  | 19. | #4 AND #11 AND #18 |

**Supplementary material**

Full Search criteria for electronic databases
